# Why GPS makes distances bigger than they are

**DOI:** 10.1080/13658816.2015.1086924

**Published:** 2015-11-16

**Authors:** Peter Ranacher, Richard Brunauer, Wolfgang Trutschnig, Stefan Van der Spek, Siegfried Reich

**Affiliations:** ^a^ Department of Geoinformatics - Z_GIS, University of Salzburg, Salzburg, Austria; ^b^ Salzburg Research Forschungsgesellschaft mbH, Salzburg, Austria; ^c^ Department of Mathematics, University of Salzburg, Salzburg, Austria; ^d^ Faculty of Architecture, Department of Urbanism, Delft University of Technology, Delft, The Netherlands

**Keywords:** GPS measurement error, trajectories, movement analysis, autocorrelation, car movement, pedestrian movement, quadratic forms

## Abstract

Global navigation satellite systems such as the Global Positioning System (GPS) is one of the most important sensors for movement analysis. GPS is widely used to record the trajectories of vehicles, animals and human beings. However, all GPS movement data are affected by both measurement and interpolation errors. In this article we show that measurement error causes a systematic bias in distances recorded with a GPS; the distance between two points recorded with a GPS is – on average – bigger than the true distance between these points. This systematic ‘overestimation of distance’ becomes relevant if the influence of interpolation error can be neglected, which in practice is the case for movement sampled at high frequencies. We provide a mathematical explanation of this phenomenon and illustrate that it functionally depends on the autocorrelation of GPS measurement error (*C*). We argue that *C* can be interpreted as a quality measure for movement data recorded with a GPS. If there is a strong autocorrelation between any two consecutive position estimates, they have very similar error. This error cancels out when average speed, distance or direction is calculated along the trajectory. Based on our theoretical findings we introduce a novel approach to determine *C* in real-world GPS movement data sampled at high frequencies. We apply our approach to pedestrian trajectories and car trajectories. We found that the measurement error in the data was strongly spatially and temporally autocorrelated and give a quality estimate of the data. Most importantly, our findings are not limited to GPS alone. The systematic bias and its implications are bound to occur in any movement data collected with absolute positioning if interpolation error can be neglected.

## 1. Introduction

Global navigation satellite systems, such as the Global Positioning System (GPS), have become essential sensors for collecting the movement of objects in geographical space. In movement ecology, GPS tracking is used to unveil the migratory paths of birds (Higuchi and Pierre 2005), elephants (Douglas-Hamilton *et al*. 2005) and roe deer (Andrienko *et al*. 2011). In urban studies, GPS movement data help detecting traffic flows (Zheng *et al*. 2011) and human activity patterns in cities (Van Der Spek *et al*. 2009). In transportation research, GPS allows monitoring of intelligent vehicles (Zito *et al*. 1995) and mapping of transportation networks (Mintsis *et al*. 2004), to name but a few application examples.

Movement recorded with a GPS is commonly stored in the form of a trajectory. A trajectory 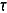 is an ordered sequence of spatio-temporal positions: 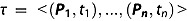, with 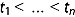 (Güting and Schneider 2005). The tuple 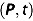 indicates that the moving object was at a position 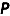 at time *t*. In order to represent the continuity of movement, consecutive positions 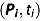 and 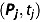 along the trajectory are connected by an interpolation function (Macedo *et al*. 2008).

However, although satellite navigation provides global positioning at an unprecedented accuracy, GPS trajectories remain affected by errors. The two types of errors inherent in any kind of movement data are measurement error and interpolation error (Schneider 1999), and these errors inevitably also affect trajectories recorded with a GPS.

Measurement error refers to the impossibility of determining the actual position 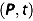 of an object due to the limitations of the measurement system. In the case of satellite navigation, it reflects the spatial uncertainty associated with each position estimate.

Interpolation error refers to the limitations on interpolation representing the actual motion between consecutive positions 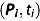 and 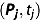. This error is influenced by the temporal sampling rate at which a GPS records positions.

Measurement and interpolation errors cause the movement recorded with a GPS to differ from the actual movement of the object. This needs to be taken into account in order to achieve meaningful results from GPS data.

In this article, we focus on GPS measurement error in movement data. We show that measurement error causes a systematic overestimation of distance. Distances recorded with a GPS are – on average – always bigger than the true distances travelled by a moving object, if the influence of interpolation error can be neglected. In practice, this is the case for movement recorded at high frequencies. We provide a rigorous mathematical explanation of this phenomenon. Moreover, we show that the overestimation of distance is functionally related to the spatio-temporal autocorrelation of GPS measurement error. We build on this relationship and develop a novel methodology to assess the quality of GPS movement data. Finally, we demonstrate our method on two types of movement data namely the trajectories of pedestrians and cars.

Section 2 introduces relevant works from previously published literature. Section 3 provides a mathematical explanation of why GPS measurement error causes a systematic overestimation of distance. Section 4 shows how this overestimation can be used to reason about the spatio-temporal autocorrelation of measurement error. Section 5 describes the experiment and presents our experimental results, Section 6 discusses the results.

## 2. Related work

Since GPS data have become a common component of scientific analyses, its quality parameters have received considerable attention. The parameters include the accuracy of the position estimate, the availability and the update rate of the GPS signal as well as the continuity, integrity, reliability and coverage of the service (Hofmann-Wellenhof *et al*. 2003). The accuracy of the position estimate (i.e. the expected conformance of a position provided with a GPS to the true position, or the anticipated measurement error) is clearly of utmost importance. Measurement error and its causes, influencing factors, and scale have been extensively discussed in published literature; measurement error has been shown to vary over time (Olynik 2002) and to be location-dependent. Shadowing effects, for example due to canopy cover, have a significant influence on its magnitude (D’Eon *et al*. 2002). Measurement error is both random, caused by external influences, and systematic, caused by the system’s limitations (Parent *et al*. 2013).

Measurement error is the result of several influencing factors. According to Langley (1997), these include:

- Propagation delay: the density of free electrons in the ionosphere and the temperature, pressure and humidity in the troposphere affect the speed of the GPS signal and hence the time that it takes to reach the receiver (El-Rabbany 2002);
- Drift in the GPS clock: a drift in the on-board clocks of the different GPS satellites causes them to run asynchronously with respect to each other and to a reference clock;
- Ephemeris error: the calculation of the ephemeris, the orbital position of a GPS satellite at a given time, is affected by uncertainties (Colombo 1986);
- Hardware error: the GPS receiver, being as fault-prone as any other measurement instrument, produces an error when processing the GPS signal;
- Multipath propagation: terrestrial objects close to the receiver (such as tall buildings) can reflect the GPS signal and thus prolong its travel time from the satellite to the receiver;
- Satellite geometry: an unfavourable geometric constellation of the satellites reduces the accuracy of positioning results.

There are several quality measures to describe GPS measurement error, the most common being the 95% radius (*R*95), which is defined as the radius of the smallest circle that encompasses 95% of all position estimates (Chin 1987). The official GPS Performance Analysis Report for the Federal Aviation Administration issued by the William J. Hughes Technical Center (2013) states that the current set-up of the GPS allows to measure a spatial position with an average *R*95 of slightly over three meters using the Standard Positioning Service (SPS). The values in the report were, however, obtained from reference stations that were equipped with high quality receivers and had unobstructed views of the sky. It is reasonable to assume that the accuracy would be reduced in other recording environments, as measurement error depends to a considerable extent on the receiver as well as on the geographic location (Langley 1997, William J. Hughes Technical Center 2013). This assumption is supported by published literature on GPS accuracy in forests (Sigrist *et al*.^1999^) and on urban road networks (Modsching *et al*. 2006), as well as on the accuracies of different GPS receivers (Wing *et al*. 2005, Zandbergen 2009). On the other hand, the accuracy of GPS can be increased using differential global positioning systems (DGPS) such as the European Geostationary Navigation Overlay Service. DGPS corrects the propagation delay caused by the ionosphere, the troposphere and the satellite orbit errors, thus yielding higher position accuracies (Hofmann-Wellenhof *et al*. 2003).

A detailed overview of current GPS accuracy is provided in the quarterly GPS Performance Analysis Report for the Federal Aviation Administration. A good introduction to the GPS in general, and to its error sources and quality parameters in particular, has been provided by Hofmann-Wellenhof *et al*. (2003).

The above-mentioned research has mainly focused on describing and understanding GPS measurement errors. In addition to this, filtering and smoothing approaches have been proposed for recording movement data in order to reduce the influence of errors on movement trajectories. A summary of these approaches can be found in Parent *et al*. (2013) and Lee and Krumm (2011). Jun *et al*. (2006) tested smoothing methods that best preserve travelled distance, speed, and acceleration. The authors found that Kalman filtering resulted in the least difference between the true movement and its representation.

## 3. GPS measurement error causes a systematic overestimation of distance

A GPS record consists of a spatial component (i.e. latitude *ϕ*, longitude 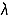) and a temporal component (i.e. a time stamp *t*). In this article we mainly focused on the spatial component.

The GPS uses the *World Geodetic System 1984 (WGS84)* as a coordinate reference system. For reasons of simplicity it is preferable to transform the GPS records to a Cartesian map projection such as the Universal Transversal Mercator (UTM). A transformation from an ellipsoid (WGS84) to a Cartesian plane (UTM) leads to a distortion of the original trajectories (Hofmann-Wellenhof *et al*. 2003). For vehicle, pedestrian, or animal movements consecutive positions along a trajectory are usually sampled in intervals ranging from seconds to minutes. Thus, these positions are very close together in space so that the distortion is insignificant for most practical applications. According to Seidelmann (1992) the distortion anywhere in a UTM zone is guaranteed to be below 1/1000. This means, for example, that the maximum distortion of a distance of 10 m is ±1 cm. Hence, for all the following considerations we can safely assume that the movement is recorded in UTM.

Very generally, a spatial position in UTM is a two-dimensional coordinate

(1) 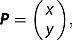

where *x* is the metric distance of the position from a reference point in eastern direction and *y* in northern direction. If a moving object is recorded at position 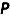 with a GPS, the position estimate 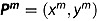 is affected by measurement error. The relationship between the true position and its estimate is trivial

(2) 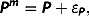

where 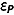 is the horizontal measurement error expressed as a vector in the horizontal plane. 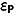 is drawn from 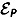, the distribution of measurement error at 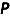. We adopted the convention used by Codling *et al*. (2008) to denote random variables with upper case letters and their numerical values with lower case letters.

**Figure 1.**
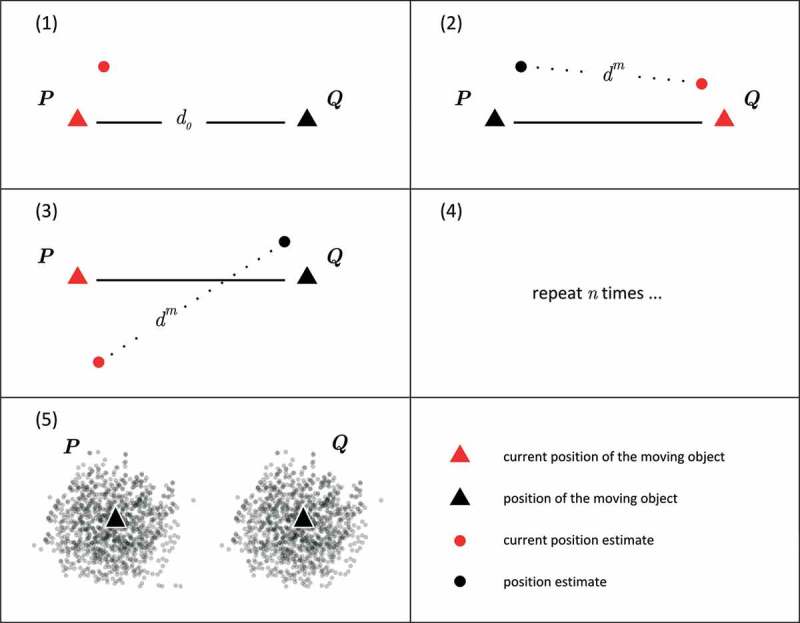
A moving object equipped with a GPS travels between two arbitrary positions.

We now provide a detailed mathematical explanation of why measurement error causes a systematic overestimation of distance in trajectories, if interpolation error can be neglected. Figure 1 illustrates the problem statement in a simplified form. Consider a moving object equipped with a GPS device. The moving object travels between two arbitrary positions 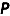 and 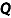. Let 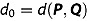 denote the Euclidean distance between these positions, henceforth referred to as reference distance. The object always moves along a straight line, consequently interpolation error can be neglected. The movement of the object can be described by the following five steps which correspond to the subplots in Figure 1.

1. The moving object starts at 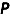. The GPS obtains the position estimate 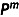 with measurement error 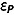, which is drawn from 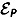.
2. The moving object travels to 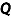. The GPS obtains the position estimate 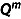 with measurement error 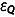, which is drawn from 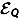. The distance between the two position estimates is calculated: 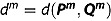.
3. The moving object returns to 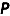. The GPS obtains a position estimate and a new 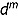 is calculated.
4. Steps 2 and 3 are repeated *n* times, where *n* is an infinitely large number.
5. After *n* repetitions, the position estimates scatter around 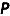 and 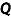 with measurement error 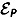 and 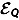.

We claim that measurement error propagates to the expected measured distance 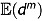 and to the expected squared measured distance 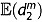 between the position estimates. More specifically, measurement error yields 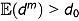 as well as 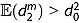.

We are now going to rigorously prove this claim. To do so, we simplify notation, write 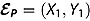 as well as 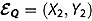, and assume that there is no systematic bias, i.e. we have 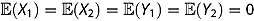. Since neither translations nor rotations affect distances between points we may, without loss of generality, consider 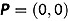 and 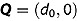. Since linear transformations (like rotations) preserve expectation, rotating errors with expectation zero results in errors having expectation zero too. Having this we can now formulate the following first result for the expected squared distance 
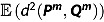
. For mathematical background we referred to Klenke (2013). Notice that no assumptions (like absolute continuity or normality) about the underlying error distributions are needed, i.e. the result holds in full generality.

Theorem 3.1: *Suppose that* 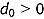, 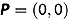, *and* 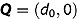. *Let* 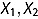 *both have distribution function F and variance* 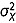, *and* 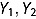 *both have distribution function G and variance* 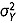. *Furthermore, assume that* 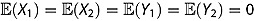, *then the following two conditions are equivalent*:

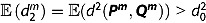

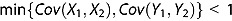

*In other words, the expected squared distance* 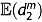 *is strictly greater than* 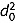 *unless the errors fulfil* 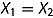 *and* 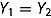 *with probability one (which describes the situation of always having identical errors in* 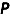 *and* 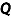).

Proof: Calculating 
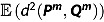
 and using the fact that 
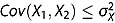
 and 
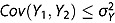
 directly yields

(3) 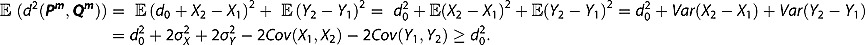

Having this it follows immediately that 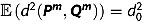 if and only if 
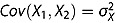
 and 
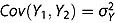
 which in turn is equivalent to the fact that 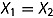 and 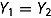 holds with probability one.▄

In general one is, however, interested in the expected distance 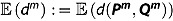 and not in the expected squared distance. Since, in general, 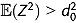 need not imply 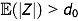 for arbitrary random variables ***Z***, a different method is used to prove the following main result

Theorem 3.2 *Suppose that the assumptions of Theorem 3.1 hold, then the following two conditions are equivalent*:

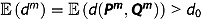

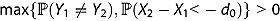

*In other words, the expected distance* 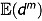 *is strictly greater than the true distance* 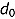 *unless the errors fulfil* 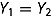 *with probability one and* 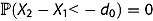 *holds*.

Proof: Obviously we have

(4) 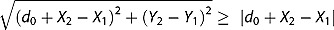

Setting 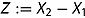 implies 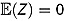. Assume now that 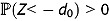 holds, then the desired inequality follows immediately from

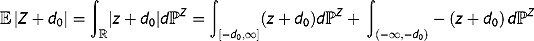

(5) 
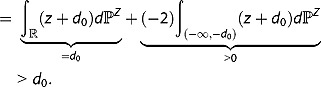

In case we have 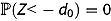 but 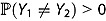 holds, then Inequality 4 is strict with probability greater than zero so we get

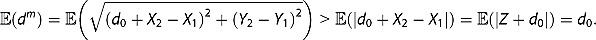

Altogether this shows that the second condition of Theorem 3.2 implies the first one.

To prove the reverse implication, assume that 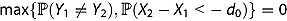. Then, firstly, the left and the right hand-sides of Inequality 4 coincide with probability one, so 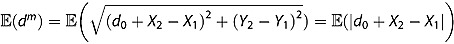 holds. And secondly, directly applying Equality 5 yields 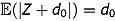, which finally shows 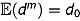.▄

Remark 3.3: *It is worth mentioning that Theorem 3.2 has several interesting (and partially surprising) consequences: Whenever the errors in x-direction are unbounded (like in the case of normal distributions) the expected distance is always strictly greater than the true distance* 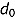. *The same holds whenever the errors* 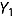 *and* 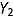 *in y-direction do not always coincide – a very realistic assumption for GPS trajectories*.

We want to underline that Theorem 3.1 and 3.2 hold in full generality for arbitrary distributions of GPS measurement error. Although GPS measurement error is often assumed to have a bivariate normal distribution and to be independent in both the *x*- and *y*-directions (Jerde and Visscher 2005, Bos*et al*. 2008), Chin (1987) puts forward convincing arguments why this is very likely not the case. Hence, the general validity of our findings is relevant.

For reasons of simplicity, we assumed that 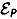 and 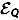 follow the same distribution function and that there is no systematic bias, i.e. 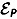 is centred around 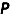 and 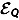 around 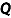. This assumption is generally acknowledged for in the literature. It builds, for example, the basis for algorithms to extract road maps from GPS tracking data (e.g. Wang *et al*. 2015). Roads are assumed to be located where the density of the GPS position estimates is the highest. Also Figure 4 shows that this assumption is indeed realistic for real-world GPS data. However, even a systematic bias does not necessary restrict the validity of our argument. Let us assume that 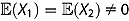 and 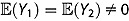, i.e. the mean of the error distribution has shifted away from 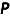 and 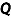 respectively. As the shift is the same for 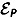 and 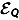, the influence on distance calculations cancels out, Theorem 3.1 and 3.2 still hold. The validity of our proof is restricted only if 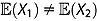 or 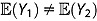. This implies that the mean of the error distribution changes abruptly between 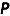 and 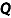. As – in practice – 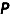 and 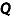 are very close in space, this scenario is not realistic for GPS measurement error.

## 4. How big is the overestimation of distance and why is this relevant?

In the previous section we proved that distances recorded with a GPS are on average bigger than the distances travelled by a moving object, if interpolation error can be neglected. In this section we provide an equation for 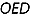, the expected overestimation of distance. Moreover, we identify three parameters that influence the magnitude of 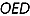. First, let us define 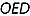 with the help of Equation (3):

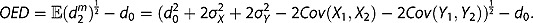

**Figure 2.**
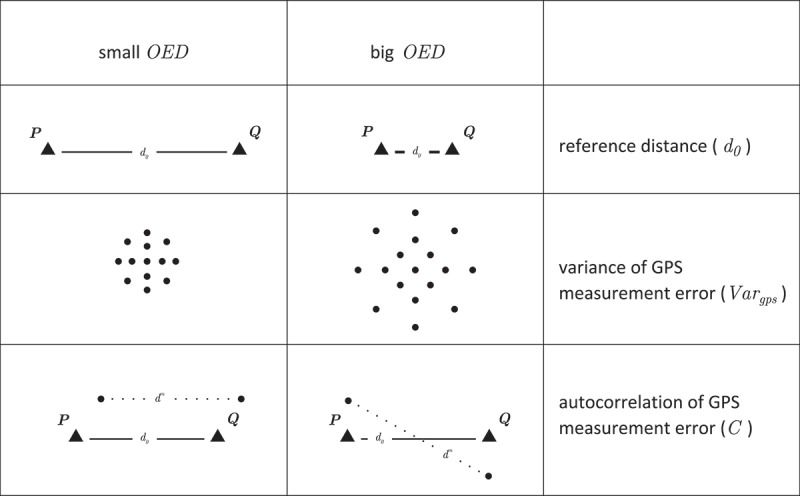
Overestimation of distance (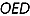) and its influencing parameters.

From this follows that 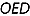 is a function of three parameters:

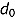
, the reference distance between 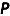 and 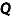

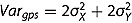
, a term for the variance of GPS measurement error

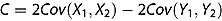
, a term for the spatiotemporal auto-correlation of GPS measurement error. *C* expresses the similarity of any two consecutive position estimates. If *C* is big, consecutive position estimates have similar GPS measurement error (see also Figure 2).

We can now simplify notation and write

(6) 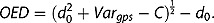

The influence of the three parameters on 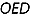 is further illustrated in Figure 2. 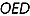 is small if the reference distance is big, the variance of GPS measurement error is small and the error has high positive spatio-temporal autocorrelation. 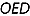 is big if the reference distance is small, the variance of GPS measurement error is big and the error has high negative autocorrelation.

**Figure 3.**
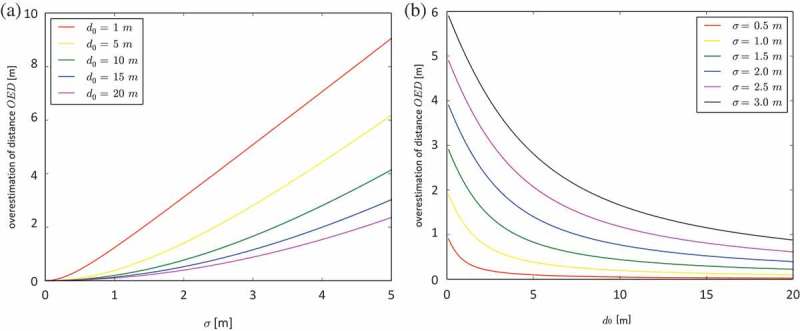
The overestimation of distance 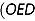) increases as the spread of GPS measurement error (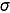) increases, the reference distance (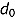) is constant (a); 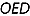 decreases as 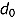 increases and 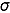 is constant (b).

To understand the magnitude of 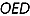 in real-world GPS data, let us assume for a moment that there is no spatio-temporal autocorrelation of GPS measurement error, i.e. 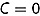. Moreover, let us assume that the variance of error is the same in *x*- and *y*-directions, i.e. 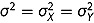 and 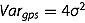. We can now visualise the relationship between 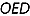, 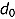 and 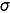. Figure 3a shows that 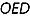 increases as the spread of GPS measurement error (σ) increases; *d*_0_ is assumed to be constant. For a constant *d*_0_ f 5 m, for example, and 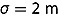, the overestimation of distance roughly equals 2 m (yellow line). When 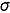 increases to 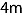, the overestimation of distance increases to 4 m. Figure 3b shows that 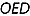 decreases as *d*_0_ increases, 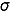 is assumed to be constant. For a constant 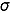 of 3 m, for example, and 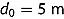, the overestimation of distance equals around 3 m (black line). When *d*_0_ increases to 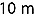, the overestimation of distance decreases to 2 m.

Remember that Figure 3 shows the influence of 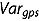 if there is no autocorrelation of GPS measurement error. This is not very realistic for real world GPS data. In fact, El-Rabbany and Kleusberg (2003), Wang *et al*. (2002) and Howind *et al*. (1999) show that GPS measurement error is temporally and spatially autocorrelated. This means that position estimates taken close in space and in time tend to have similar error.

How big is the autocorrelation of GPS measurement error? Let us reformulate Equation (6) and solve for *C*:

(7) 
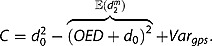

This implies that we can calculate the autocorrelation of GPS measurement error if 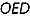, 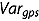 and 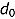 are known. Things become interesting if we consider what autocorrelation really means in the context of GPS positioning. In Figure 2, in the bottom left cell, the position estimates 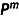 and 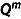 are highly autocorrelated and, hence, very similar. This leads to the effect that 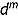 is very similar to 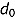. In fact, this applies not only to distance, but also to other movement parameters as well. Direction, speed, acceleration or turning angle must all be similar to the ‘true’ movement of the object if they are derived from highly autocorrelated GPS position estimates. Consequently, *C* describes how well a GPS captures the movement of an object, if interpolation error can be neglected. Or in other words, *C* is a quality measure for GPS movement data.

## 5. Assessing the quality of GPS movement data

**Figure 4.**
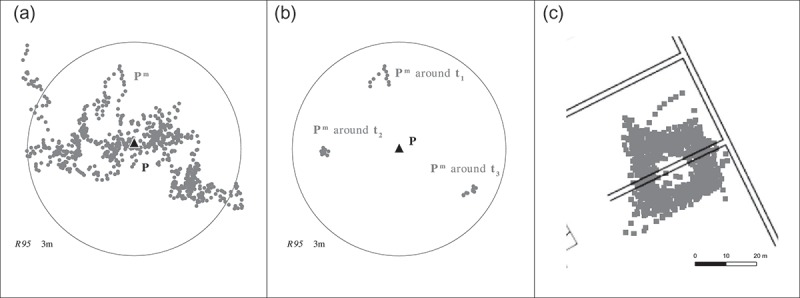
The distribution of GPS measurement error at position 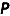 (a). Revealing the temporal autocorrelation of GPS measurement error (b). The movement of a pedestrian around a reference course (c).

Real world GPS data are temporally and spatially autocorrelated (Howind *et al*. 1999, Wang *et al*. 2002, El-Rabbany and Kleusberg 2003). Spatial autocorrelation implies that GPS measurement error is not independent of space. Position estimates obtained at similar locations will have similar error. Temporal autocorrelation implies that GPS measurement error is not independent of time. Position estimates obtained at similar times will have a similar error due to similar atmospheric conditions and a similar satellite constellation (Bos *et al*. 2008). We carried out a simple experiment to visualise temporal autocorrelation in real-world GPS data. We placed a GPS logger at a known position 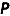 and recorded about 720 position estimates over a period of about six hours at a sampling rate of 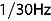. The resulting distribution is centred around 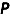 with an 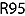 of about 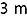 (Figure 4a). If only those position estimates are displayed that were recorded within a certain time interval, GPS measurement error reveals itself to be highly auto-correlated. Figure 4b, for example, shows only those position estimates that were obtained within periods covering 5 minutes before and after 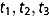.

In this section we build on the relationship described in Equation (7) and show the spatial and temporal autocorrelation in two sets of real-world GPS movement data. In the first experiment we identified to what degree a set of pedestrian movement data was temporally and spatially autocorrelated. In the second experiment we derived the spatial autocorrelation in a set of car movement data. Based on this we tried to assess how well the GPS captured the movement of the car.

### 5.1. Experiment 1: pedestrian trajectories

#### 5.1.1. Experimental setup

For the first experiment, we equipped a pedestrian with a GPS. The pedestrian walked along a reference course with a well-established reference distances 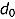. The movement of the pedestrian was recorded with a QSTARZ:BT-Q1000X GPS logger^1^ with ‘Assisted GPS’ activated.

Rather than using a high-quality GPS we collected all data with a low-budget GPS, a type of GPS common for recording movement data. We deliberately treated the GPS as a ‘black box’. This implies that the algorithm to calculate the position estimates from the raw GPS signal was not known. Moreover, we considered that it was sufficient to use only a single GPS logger, as the aim of the experiment was not to investigate the quality of the particular GPS, but to show the usefulness of our approach.

The reference course was located in an empty parking lot to avoid shadowing and multi-path effects. We staked out a square with sides that were 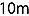 long. We placed markers along the sides of the square at one meter intervals using a measuring tape. The square allowed us to collect distance measurements approximately in all four cardinal directions. The distance between the markers was used as a reference distance 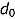.

The GPS position estimates were obtained by walking to the reference markers in turn and recording the position, moving around the square until all positions of the markers had been recorded. Position estimates were only taken at the reference markers, and only when the recording button was pushed manually. Two consecutive position estimates were taken within three to five seconds. A full circuit around the square took approximately between two and three minutes and resulted in 40 positions being recorded. A total of 25 circuits around the square were completed without any breaks. This resulted in 1000 GPS positions being collected in approximately one hour. A first extra circuit around the square was not considered for analysis to account for possible large errors after the cold start of the GPS device.

In pre-processing, distance measurements 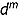 were calculated between the position estimates and later compared with 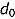 the reference distance between the markers. Then the average measured distance 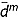 was calculated and from this 
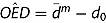
 and 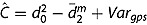 were derived. 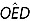 and 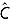 are estimators for 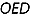 and *C*.

We set 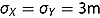. These values were not directly calculated from empirical measurements, but rather based on our experience with the particular GPS device. Hence, 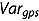 is not the observed variance of GPS measurement error, but a reference value to which 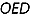 is later compared with. Consequently, our results do not show the exact value of *C*, but provide an estimate of *C* with respect to 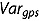.

We increased the spatial separation between two position estimates of the pedestrian to illustrate the influence of spatial autocorrelation. Then we increased the temporal separation between two position estimates to illustrate the influence of temporal autocorrelation.

#### 5.1.2. Results

**Figure 5.**
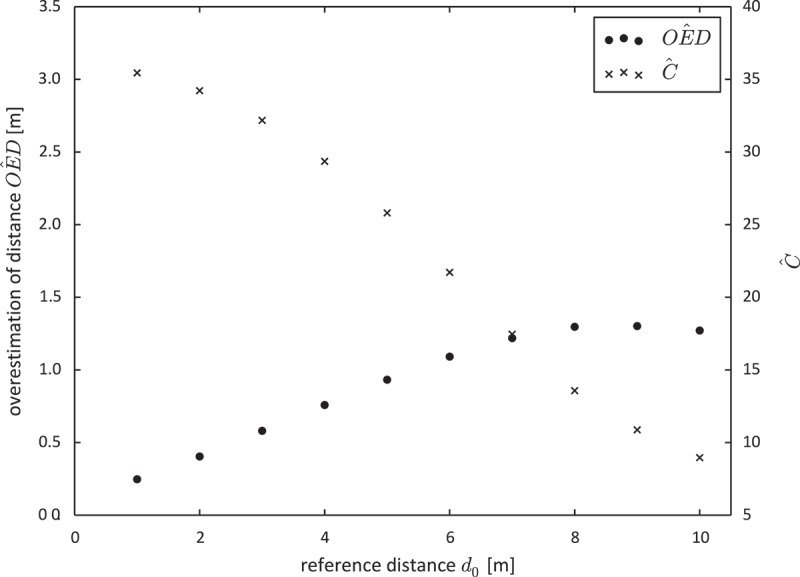
Overestimation of distance (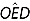) and spatial autocorrelation of GPS measurement error (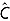) in the pedestrian movement data.

In contrast to the theoretical findings in Figure 3, overestimation of distance tended to increase as the reference distance 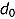 increased. This was due to a decrease in the spatial autocorrelation of GPS measurement error. With increasing spatial separation of the position estimates, measurement error became less autocorrelated. Figure 5 shows the relationship between the reference distance 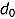 and 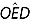 (black dots) as well as 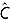 (black crosses).

**Figure 6.**
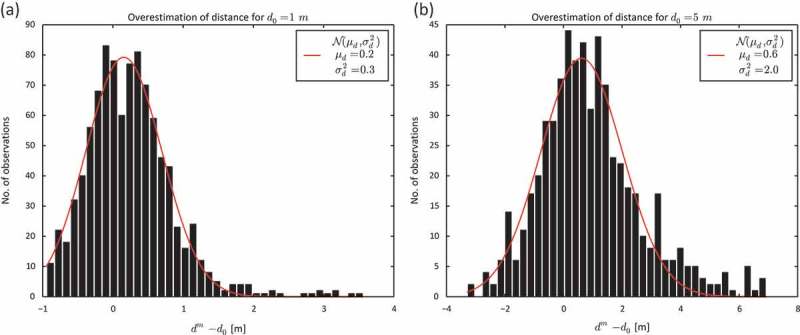
Histogram of the difference between measured and reference distance (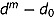) for 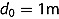 (a) and 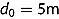 (b).

We wanted to illustrate that the overestimation of distance was not caused by a small number of extreme outliers. Figure 6 shows the histogram of 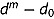 for 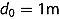 (a), and for 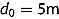 (b) and their fit to a Gaussian distribution. Both histograms follow a Gaussian distribution 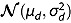 rather well and outliers are almost non-existent. Note that 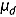 and 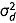 in Figure 6 refer to the values of the fitted Gaussian distribution and not to the empirically derived frequency.

**Figure 7.**
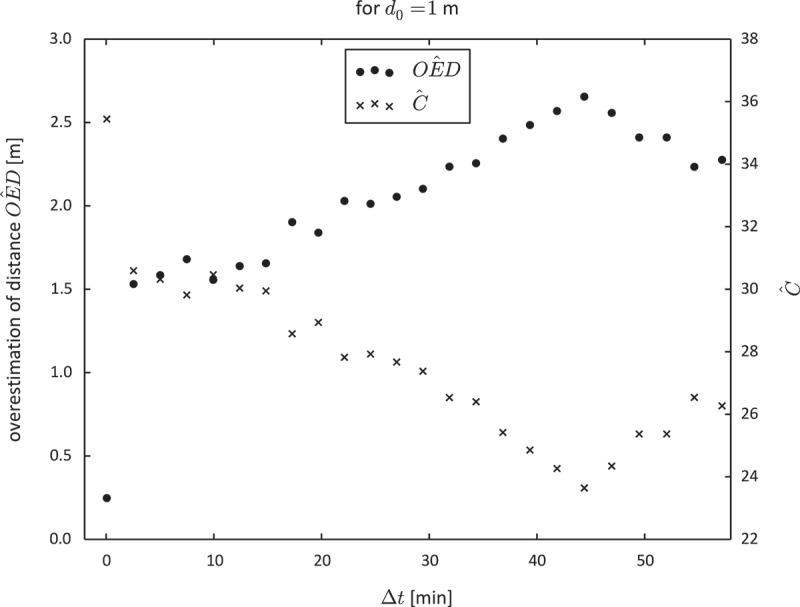
Overestimation of distance 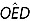 and temporal autocorrelation of GPS measurement error (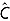) in the pedestrian movement data.

In order to illustrate the temporal autocorrelation in GPS measurement error, we calculated the distance between non-consecutive position estimates around the square. One example is the distance between two position estimates, where the second one was obtained one circuit after the first. The reference distance between the markers remained the same, e.g. 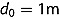, but the position estimates were recorded within a longer time interval 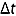. Figure 7 shows the relationship between 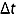 and 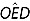 (black dots) as well as 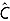 (black crosses) for a reference distance 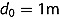. 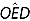 increases with longer time intervals. The sharpest increase occurs between position estimates that were taken promptly and those taken after about 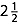 minutes. After 40 minutes the curve levels out. This increase of 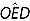 was caused by the temporal autocorrelation of measurement error. For position estimates taken within several seconds, measurement error appears to be strongly autocorrelated. However, autocorrelation falls sharply for position estimates taken within 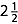 minutes. From then on 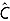 gradually decreases as 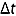 increases; again the curve levels out at about 40 minutes.

The data for the above experiment were calculated with a GPS for which the algorithm to calculate the position estimates from the raw GPS signal was not known. This raises the legitimate question whether the results were produced by a smoothing algorithm rather than the behaviour of the GPS. Let us assume that the GPS used a smoothing algorithm. In simplified form, the current position estimate is then calculated from the last position estimate, the current GPS measurement and a movement model. For movement with constant speed and direction, smoothing yields trajectories that represent the true movement very accurately. However, sudden changes in movement, i.e. a sharp turn, are not followed by the trajectory. The current measurement implies a sharp turn, however, the movement model does not. Thus, the sharp turn becomes more elongated, the overestimation of distance increases. However, we did not find any support for an increase in the overestimation of distance after a sharp turn. This can also be seen in Figure 4b.

### 5.2. Experiment 2: car trajectories

In the first experiment the reference distance 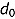 was staked out along a reference course. For obvious reasons this is not possible for recording the movement of a car. Hence we derived 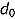 from speed measurements recorded with a car’s controller area network bus (CAN bus).

#### 5.2.1. Experimental setup

We equipped a car with a GPS logger and tracked its movement for about 6 days. The car moved mostly in an urban road network at rather low speeds (average: 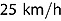). The temporal sampling rate of recording was 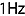. For the CAN bus measurements, a sensor recorded the rotation of the car’s drive axle, from which 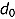 was inferred. Thus 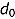 is the distance travelled by the car according to the CAN bus. For the same phases of movement we compared 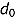 with 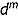, the distance travelled by the car according to the GPS position estimates. As in the first experiment, we set 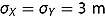 and calculated 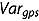.

The data were first pre-processed and cleaned. Parts were removed where the data suggested that the car had considerably exceeded the Austrian speed limit (above 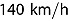) or that it had moved at a physically not realistic acceleration (above 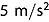). Although the data consisted mostly of the car’s forward movements, there were also periods when it was either stationary or reversing in a parking lot. The data may also have included some periods during which shadowing caused a loss of the GPS signal (for example when driving in a tunnel). We therefore applied a simple mode detection algorithm to remove any such periods. The algorithm evaluates speed and acceleration along the trajectory and distinguishes segments that most probably reflect driving behaviour from those that are likely to reflect non-driving behaviour (Zheng *et al*. 2010). Using the algorithm we were able to include only long phases of continuous driving, sampled at a continuous sampling frequency of 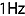. Following this pre-processing a total of about 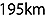 of car trajectories remained for analysis.

#### 5.2.2. Results

**Figure 8.**
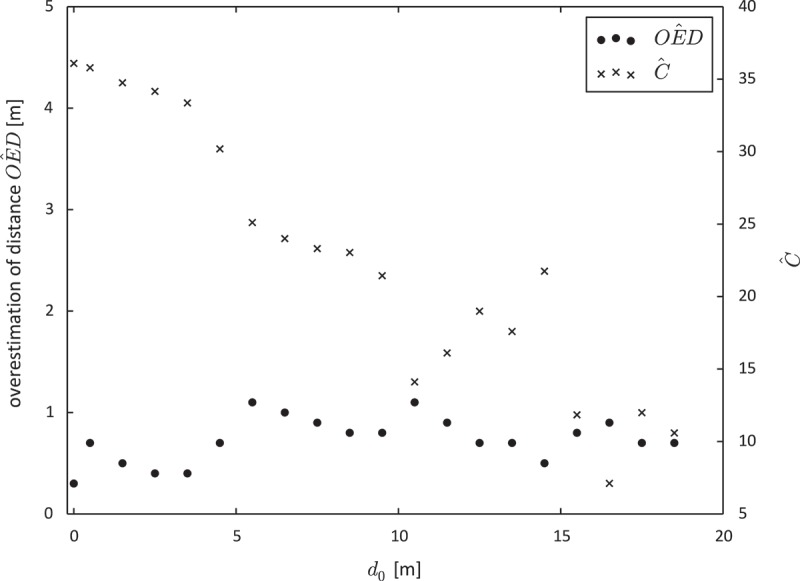
Overestimation of distance (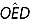) and spatial autocorrelation of GPS measurement error (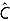) in the car movement data.

Figure 8 shows that the autocorrelation of GPS measurement error decreased as the spatial separation between two consecutive position estimates increased. Nevertheless, 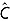 in Figure 8 is always positive. This can be interpreted as a quality measure for the movement data. Consecutive position estimates have less variance than initially suggested by 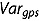.

Although the results in Figure 8 are similar to those obtained from the pedestrian movement data (see Figure 5), they contain outliers. We believe that these outliers occur due to two reasons. First, the data comprise relatively few distance measurements for big 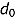 because of the generally low speed of the car. Second, we could not guarantee a full temporal synchronisation of both measurement systems (GPS and CAN bus). In other words, 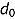 and 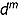 might relate to slightly different time intervals. We found this lag to be around one second. We believe that this insight is important for the practical application of Equation (7). In order to provide valid results it requires both a significant number of distance measurements as well as a proper synchronisation of reference and measured distance.

## 6. Discussion and outlook

In this article we identified a systematic bias in GPS movement data. If interpolation error can be neglected GPS trajectories systematically overestimate distances travelled by a moving object. This overestimation of distance has previously been noted in the trajectories of fishing vessels (Palmer 2008). For high sampling rates the distance travelled by the vessel was overestimated due to measurement error, while for lower sampling rates it was underestimated due to the influence of interpolation error. We provided a mathematical explanation for this phenomenon and showed that it functionally depends on three parameters, of which one is *C*, the spatio-temporal autocorrelation of GPS measurement error. We built on this relationship and introduced a novel approach to estimate *C* in real-world GPS movement data. In this section we want to discuss our findings and show their implications for movement analysis and beyond.

In the era of big data, more and more movement data are recorded at finer and finer intervals. For movement recorded at very high frequencies (e.g. 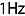) interpolation error can usually be neglected. Hence 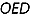 is bound to occur in these data. However, this does not mean that high frequency movement data are of low quality, quite the opposite is true. Using the relationship between *C* and 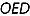 we showed experimentally that GPS measurement error in real world trajectories is temporally and spatially autocorrelated. In other words, if the data were recorded close in space and time they captured the movement of the object better than if they were further apart.

Autocorrelation is important for movement analysis in many aspects. An appropriate sampling strategy for recording movement data, for example, should consider the influence of measurement error and address spatial and temporal autocorrelation. Since autocorrelation can be interpreted as a quality measure, it allows to reveal the performance of different GPS receivers in different recording environments. Moreover, autocorrelation has implications for simulation. Laube and Purves (2011) performed a simulation to reveal the complex interaction between measurement error and interpolation error and their effects on recording speed, turning angle and sinuosity. Their Monte Carlo simulation assumed GPS errors to scatter entirely randomly between each two consecutive positions. Our approach allows to verify whether this assumption is realistic.

One might also view at the mathematical relationship discussed in the article from a different perspective. If the variance and the spatio-temporal autocorrelation of a GPS device in a particular recording environment are known, one is able to calculate the expected overestimation of distance in the trajectory data. This information can be used to give a more realistic estimate of the distance that a moving object has travelled.

### 6.1. Where to find a reference distance?

For practical applications the biggest limitation of our experiments is their dependency on a valid reference distance. The moving object must traverse the reference distance along a straight line and without interpolation error, and at a precisely known time. Moreover, a large number of position estimates has to be collected, since *C* is derived from the expectation value of a random variable.

This limitation leads to a possibly interesting application of our findings, where the reference distance is derived from the GPS point speed measurements. Point speed measurements are calculated from the instantaneous derivative of the GPS signal using the Doppler effect. Point speed is very accurate (Bruton*et al*. 1999) and usually part of a GPS position estimate. Hence, for high sampling rates (e.g. 1 Hz) point speed measurements can be used to infer the distance that a moving object has travelled between two position estimates. This distance is not affected by the overestimation of distance effect and could serve as a reference distance. Thus, GPS could be compared with itself to reveal the spatio-temporal autocorrelation of the position estimates. This approach would not require any other ground truth data, however, its feasibility and usefulness are yet to be tested.

Our findings are not only relevant for GPS. The overestimation of distance is bound to occur in any type of movement data where distances are deduced from imprecise position estimates, of course only if interpolation error can be neglected.

## Disclosure statement

No potential conflict of interest was reported by the authors.

## Note

1. For specifications, please refer to: http://www.qstarz.com/Products/GPS/20Products/BT-Q1000.html.
